# Syncope Associated with Carbon Monoxide Poisoning due to Narghile Smoking

**DOI:** 10.1155/2013/796857

**Published:** 2013-03-25

**Authors:** Seda Ozkan, Tayfun Ozturk, Yavuz Ozmen, Polat Durukan

**Affiliations:** Department of Emergency Medicine, Erciyes University Medical School, 38039 Kayseri, Turkey

## Abstract

Narghile smoking is a traditional method of tobacco use, and it has been practiced extensively for 400 years. Traditionally, narghile smoking is a matter of culture mainly in Middle East, Asia, and Africa. In recent years, its use as a social activity has increased worldwide, especially among young people. Narghile smoking is an unusual cause of carbon monoxide poisoning. Narghile smoking, compared to cigarette smoking, can result in more smoke exposure and greater levels of carbon monoxide. We present an acute syncope case of a 19-year-old male patient who had carbon monoxide poisoning after narghile smoking.

## 1. Introduction

Carbon monoxide (CO) intoxication can be notoriously nonspecific in its initial presentation to the emergency department (ED), leading to a delay in diagnosis and treatment [1, 2]. The well-known sources of CO poisoning are home heaters, car exhaust, hot water heaters, portable generators, fireplaces, tobacco smoke, and so forth. Narghile smoking is an unusual cause of CO poisoning. Narghile is known by a number of different names, including argileh, goza, hookah, hubblebubble, and shisha [3]. Traditionally, narghile smoking is a matter of culture mainly in Middle East, Asia, and Africa. It has been recently spreading to European countries and USA [1]. Narghile smoking is a kind of social event in café especially among young people in Turkey. We present an acute syncope case of a 19-year-old male patient who had carbon monoxide poisoning after narghile tobacco smoking.

## 2. A Case Report

A 19-year-old male patient was brought to our emergency department with ambulance after narghile smoking. He presented to our ED with dizziness, syncope, and blurred vision. When he arrived to ED, he was conscious. In his medical history, he had nothing important, and he was not a cigarette smoker. His friend told us that he smoked two narghile at a narghile café in about 4–5 hours, and, after standing to go home he had blurring of vision and dizziness. He did not remember how he came to the hospital.

His vital signs revealed a temperature of 36°C, blood pressure of 135/69 mmHg, respiratory rate of 20/min, pulse rate of 88/min, and pulse oximetry reading of 97% on room air. He did not complain about chest pain, but he had nausea. Physical examination revealed no abnormality.

Bedside blood glucose level was 114 mg/dL. In the patient's ECG, there was a T-wave inversion at DIII and aVF derivations. His initial COHb level was 32.7%. He was treated with 100% normobaric oxygen by a nonrebreather mask. The patient had normal biochemical examinations results including complete blood count, electrolytes, cardiac enzymes, and troponin I. At the fourth hour of treatment, his COHb level was decreased to 9.5%, and, at discharge, it was 0.3%. The patient's control cardiac enzymes and troponin I levels were all normal. His ECG did not show any change. His echocardiography revealed normal ejection fraction and no segmental cardiac wall motion abnormalities.

The patient was monitorized and followed up for 18 hours at ED. His initial clinical symptoms and COHb levels were normalized, and he was discharged from ED with complete recovery.

## 3. Discussion

**Figure 1:**
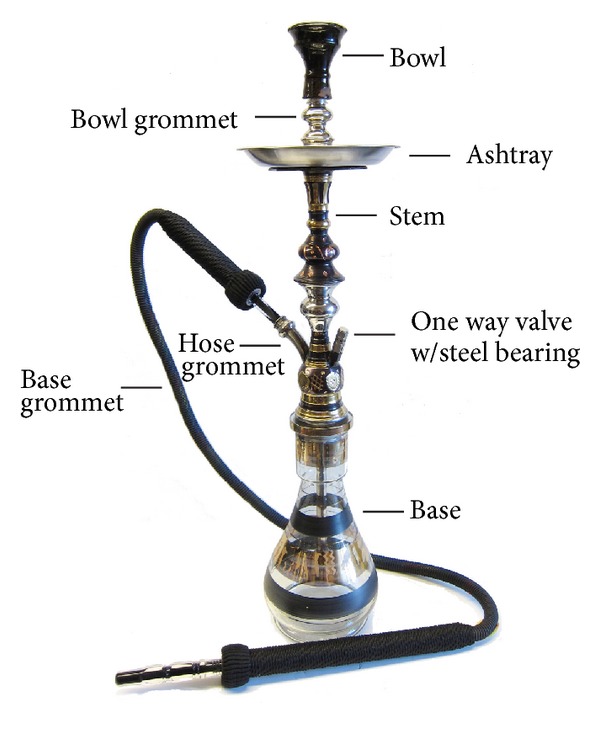
Narghile.

The narghile apparatus consists of a base that is filled with water, a bowl, a heating device that contains the tobacco, a pipe that connects the bowl to the base, and a hose that is attached to the base to allow the smoke to be inhaled. When the smoker inhales through the hose, the smoke from the tobacco passes through the water into a chamber and then is inhaled. The composition of the tobacco used in narghile is variable and not well standardized (Figure 1) [3].

Studies that have examined narghile smokers and the aerosol of narghile smoke have reported high concentrations of CO, nicotine, “tar,” and heavy metals. These concentrations were as high as or higher than those among cigarette smokers [3]. It has been estimated that smoke exposure could be as much as 100–200 cigarettes per session [2]. Narghile smokers absorb higher concentrations of CO than cigarette smokers because of the larger volumes inhaled with each puff and the longer duration of smoking session. CO concentrations in the inhaled vapours are higher because of the charcoal used to burn the narghile tobacco [4]. 

As we evaluated limited number of cases in the literature, we saw that after narghile smoking patients come to ED with nonspecific symptoms like nausea, vomiting, headache, weakness, vertigo, presyncope, and syncope. We noticed that cases are all social smokers at cafés between the ages of 16 and 27 [1, 2, 4, 5]. Our case was also a social smoker of narghile. We think that because he heavily smoked it for many hours, the event resulted in syncope.

In all the reported cases identified, COHb levels at presentation to hospital ranged from 20% to 30%. All patients were treated with oxygen supply and did well clinically [1, 2, 4, 5]. The initial COHb level of our case was also similar to the literature, 32.7%. He was treated with 100% normobaric oxygen by a nonrebreather mask. COHb level of our patient decreased to normal level (0.3%) after treatment. 

As a conclusion, increased popularity of narghile smoking in the recent years in especially young population will increase the number of cases of CO poisoning cases with nonspecific symptoms. So, it must be kept in mind that patients with nonspecific symptoms should remind us to CO poisoning, even narghile smoking.
